# Systemic inflammation response index as a prognostic predictor in patients with acute ischemic stroke: A propensity score matching analysis

**DOI:** 10.3389/fneur.2022.1049241

**Published:** 2023-01-10

**Authors:** Hui Dang, Wenjuan Mao, Shanshan Wang, Jing Sha, Mingjia Lu, Li Cong, Xuegang Meng, Hongyan Li

**Affiliations:** ^1^Department of Neurology, People's Hospital of Xinjiang Uygur Autonomous Region, Urumqi, China; ^2^Xinjiang Clinical Research Center for Stroke and Neurological Rare Disease, Urumqi, China; ^3^Department of Respiratory and Critical Care Medicine, People's Hospital of Xinjiang Uygur Autonomous Region, Urumqi, China

**Keywords:** systemic inflammation response index, ischemic stroke, predictor, mortality, propensity score matching (PSM)

## Abstract

**Background:**

Acute ischemic stroke (AIS), the most common type of stroke, is a major cause of morbidity and mortality worldwide. A growing number of studies have demonstrated that inflammation is a critical mechanism in AIS. Being an easily available and effective inflammatory marker, the systemic inflammation response index (SIRI) shows a high association with mortality in patients with cancer and intracerebral hemorrhage. In this study, we evaluated the potential prognostic role of SIRI in critically ill patients with AIS.

**Methods:**

Clinic data were extracted from the Medical Information Mart data for the Intensive Care IV (MIMIC-IV) database. The optimal cutoff value of SIRI was determined by X-tile software. The primary outcome was the 90-day all-cause mortality, and the secondary outcomes were 30-day and 1-year all-cause mortality of patients with AIS. Cox proportional hazards regression analyses were used to assess the association between SIRI levels and all-cause mortality, and survival curves were estimated using the Kaplan–Meier method. Furthermore, a 1:1 propensity score matching (PSM) method was performed to balance the influence of potential confounding factors.

**Results:**

A total of 2,043 patients were included in our study. X-tile software indicated that the optimal cutoff value of the SIRI for 90-day mortality was 4.57. After PSM, 444 pairs of score-matched patients were generated. Cox proportional hazard model showed that after adjusting for possible confounders, high SIRI level (≥4.57) was independently associated with the 90-day all-cause mortality in the cohort before PSM (HR = 1.56, 95% CI: 1.30–1.89, *p* < 0.001) and the PSM subset (HR = 1.47, 95% CI: 1.16–1.86, *p* = 0.001). The survival curves showed that patients with SIRI ≥4.57 had a significantly lower 90-day survival rate in the cohort before PSM (56.7 vs. 77.3%, *p* < 0.001) and the PSM subset (61.0 vs. 71.8%, *p* = 0.001). Consistently, AIS patients with high SIRI levels (≥4.57) presented a significantly high risk of 30-day and 1-year all-cause mortality before and after PSM.

**Conclusion:**

A higher SIRI (≥4.57) was associated with a higher risk of 90-day, 30-day, and 1-year mortality and was an independent risk factor of mortality in patients with acute ischemic stroke.

## Introduction

Stroke is an acute cerebrovascular disease caused by sudden rupture or blockage of blood vessels in the brain. It is the second leading cause of death and the third leading cause of death and disability combined in the world (1). Acute ischemic stroke (AIS) is the most common type of stroke, accounting for ~84.4% of prevalent strokes (2). Due to an aging society, the burden of stroke increases the pressure on patients, their families, and society, especially among Intensive Care Unit (ICU) patients. It is critical to identify prognostic factors for predicting patients with the highest risk of adverse outcomes. To be considered useful, risk markers, which are simpler and less expensive, should provide incremental information and possess a clear pathophysiological basis for further therapy.

Growing evidence has indicated that inflammation is a key mechanism underlying the pathophysiological processes of brain injury during various stages of cerebral ischemic injury. It is one of the crucial contributors to secondary brain injury induced by leukocyte infiltration, blood–brain barrier impairment, secretion of multiple inflammatory mediators, brain edema, and neuronal cell death (3, 4). Besides changes in the brain, AIS also induces severe extra-cerebral pathophysiological alterations, including autonomic dysfunction, activation of the hypothalamic–pituitary–adrenal (HPA) axis, and systemic immune dysregulation, which induce or aggravate functional impairment in multiple peripheral organs (5). As a result, both the brain and peripheral organs are challenged due to sustained exposure to chronic low-grade inflammation induced by AIS (6).

As inflammatory indicators, some risk markers composed of ratios of white blood cell (WBC) subgroups have been widely used in clinical practice, such as neutrophil-to-lymphocyte ratio (NLR), lymphocyte-to-monocyte ratio (LMR), and platelet-to-lymphocyte ratio (PLR). These indicators are easily available and are considered to be associated with increased risk of AIS (7–9), as well as intracranial atherosclerotic stenosis (10), coronary artery disease (11), cardiac arrest (12), and overall death (13).

The systemic inflammation response index (SIRI) is a novel prognostic marker based on the composition ratio of peripheral neutrophil, monocyte, and lymphocyte counts (calculated by neutrophil count × monocyte count/lymphocyte count). In previous studies, SIRI was found to be an independent prognostic indicator in cancer (14), rheumatoid arthritis (15), hyperuricemia, mechanical thrombectomy for large artery occlusion (16), and intracerebral hemorrhage (17). Few retrospective observational studies have analyzed the value of SIRI in AIS (18). In this study, we explored the relationship between SIRI and the risk of mortality in patients with AIS based on the Medical Information Mart for Intensive Care (MIMIC)-IV database.

## Materials and methods

### Data source

All data used in our study were derived from the Medical Information Mart for Intensive Care IV (MIMIC-IV 2.0) database. The MIMIC-IV database is a large, real-world, and publicly available clinical database established by Beth Israel Deaconess Medical Center in Boston, MA, USA, from 2012 to 2019. It includes demographic data, vital signs, laboratory parameters, and other treatment information. Furthermore, it provides the accurate date of death in the hospital or 1 year after discharge, which makes it possible for clinicians to conduct prognostic-related research. Our right to access the database was approved by the Institutional Review Board of the Massachusetts Institute of Technology (Cambridge, MA, USA) after completing online training from the Collaborative Institutional Training Initiative (CITI) program of the National Institutes of Health (NIH) (Record ID 38792967).

### Patient selection

The patients of the present study were all recruited from the MIMIC-IV database, according to the following inclusion criteria: (1) patients diagnosed with AIS based on the ninth revision of the International Classification of Diseases (ICD-9) code and ICD-10 code; (2) adult patients aged 18 years and older; and (3) the first admission to intensive care units (ICU). Patients who met one of the following criteria were excluded: (1) absence of SIRI results within the first 24 h of admission; and (2) patients who suffered from hematologic neoplasms.

### Data extraction

The data were extracted from MIMIC-IV using Structured Query Language (SQL), and pgAdmin4 for PostgreSQL was used as the administrative platform. The extracted data included: (1) demographics: age, gender, and ethnicity; (2) comorbidities: hypertension, dyslipidemia, history of stroke, congestive heart failure (CHF), peripheral vascular disease, chronic pulmonary disease, diabetes mellitus, malignant cancer, severe liver disease, and atrial fibrillation; (3) laboratory parameters: white blood cell (WBC) count, neutrophil count and percentage, monocyte count and percentage, lymphocyte count and percentage, platelet count, hemoglobin, serum glucose, serum creatinine, serum sodium, serum potassium, serum anion gap, prothrombin time (PT), activated partial thromboplastin time (APTT), NLR, PLR, LMR, and SIRI; (4) metrics including Sequential Organ Failure Assessment (SOFA) and Glasgow Coma Scores (GCSs); and (5) treatment including thrombolysis and thrombectomy. Clinical outcomes include the length of ICU stay (LOS ICU), length of hospital stay (LOS hospital), 90-day all-cause mortality, 30-day all-cause mortality, and 1-year all-cause mortality. The primary outcome was the 90-day all-cause mortality. The secondary outcomes were the 30-day mortality and 1-year all-cause mortality of patients with AIS.

### Propensity score matching

Due to the retrospective design of the study, the patient selection criteria were less likely to be random. Thus, propensity score matching (PSM) analysis was performed to reduce the influence of selection bias and potential confounding factors. PSM analysis was based on a logistic regression model, and the propensity scores were examined according to the following variables: age, gender, ethnicity, hypertension, dyslipidemia, history of stroke, CHF, peripheral vascular disease, chronic pulmonary disease, diabetes mellitus, malignant cancer, severe liver disease, atrial fibrillation, WBC count, platelet count, hemoglobin, serum glucose, serum creatinine, serum sodium, serum potassium, anion gap, PT, APTT, SOFA, and GCS score. The PSM analysis was conducted using a 1:1 nearest neighbor matching algorithm with a caliper of 0.1. We evaluated the balance between the two groups by using the absolute standardized differences (ASDs) before and after matching. ASDs <0.10 implied the characteristics to be well-balanced.

### Statistical analysis

Continuous variables were presented as mean ± SD or median (interquartile range) and compared using the *t*-test or Mann–Whitney *U*-test. Categorical variables were expressed as total numbers with proportions and analyzed using the Chi-square test or Fisher's exact test. The optimal cutoff value of the SIRI for 90-day mortality was determined by X-tile (Version 3.6.1, Yale University School of medicine) software. SIRI was divided into two groups using predefined optimal cutoff values for further analyses. In addition, SIRI was divided into four equal-interval categories to analyze the association between different SIRI quartiles and all-cause mortality. The first binary or quartile was selected as the reference group. Pearson correlation coefficient was used to analyze the correlation between the variables, with *r* < 0.7 indicating that these two variables have no relationship or correlation. Survival curves were estimated using the Kaplan–Meier method and compared by the log-rank test. The Cox proportional hazards model was applied for the univariate and multivariate analyses to identify independent prognostic factors of 90-day, 30-day, and 1-year mortality after AIS. The results are presented as hazard ratios (HRs) and 95% confidence intervals (CIs). Stratification analyses were performed to examine the effect of SIRI levels in different subgroups using a Cox regression model according to age strata (<70 and ≥70 years), gender, ethnicity, hypertension, CHF, chronic pulmonary disease, diabetes mellitus, malignant cancer, severe liver disease, and atrial fibrillation. Furthermore, the time-dependent ROC analysis was conducted to compare SIRI predictive accuracy with other blood-based inflammatory biomarkers, such as NLR, LMR, and PLR. All tests were two-sided, and *p*-values of <0.05 were considered significant. All statistical analyses in our study were performed using R statistical software (R version 4.2.2, R Foundation for Statistical Computing), SPSS Statistics 26 (IBM, Chicago, IL, USA), and GraphPad Prism 8 (GraphPad Software, San Diego, CA, USA).

## Results

### Characteristics of patients

**Figure 1:**
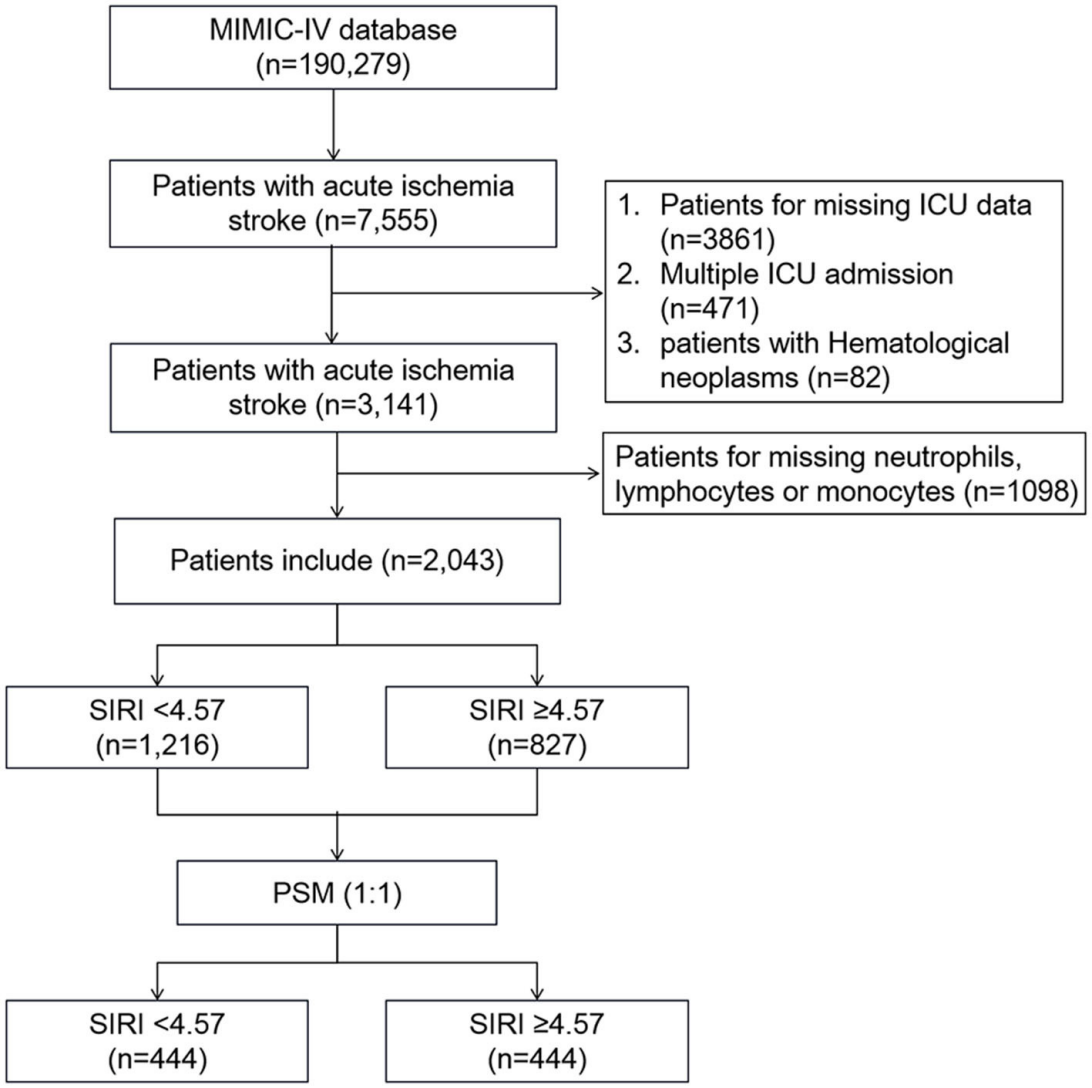
Workflow chart for the patient selection process.

A total of 2043 patients with AIS from the MIMIC-IV database who met the selection criteria were included in our study. Figure 1 shows the flowchart of the study cohort selection from the MIMIC-IV database. Patients were stratified based on the cutoff value calculated by the X-tile software. The optimal cutoff value of SIRI for 90-day mortality was set as 4.57, which divided all patients into two groups: low (<4.57) SIRI group (*n* = 1,216) and high (≥4.57) SIRI group (*n* = 827). The baseline characteristics of enrolled patients are briefly displayed in Table 1, including demographics, vital signs, laboratory parameters, comorbidities, and scores.

**Table 1:** Demographic and clinical characteristics before propensity score matching.

**Characteristic**	**Total (*n* = 2,043)**	**SIRI**		***p-*value**
		<**4.57 (*****n*** = **1,216)**	≥**4.57 (*****n*** = **827)**	
**Demographics**				
Age, years	70.4 (57.9–81.8)	69.8 (56.9–81.8)	71.3 (58.6–81.7)	0.461
Male, *n* (%)	1,029 (50.4)	625 (51.4)	404 (48.9)	0.258
Ethnicity, *n* (%)				0.002
White	1,255 (61.4)	754 (62.0)	501 (60.6)	
Black	239 (11.7)	162 (13.3)	77 (9.3)	
Others	549 (26.9)	300 (24.7)	249 (30.1)	
**Comorbidities**				
Hypertension, *n* (%)	1,531 (74.9)	909 (74.8)	622 (75.2)	0.814
Dyslipidemia, *n* (%)	1,029 (50.4)	655 (53.9)	374 (45.2)	<0.001
Previous stroke, *n* (%)	249 (12.2)	159 (13.1)	90 (10.9)	0.137
CHF, *n* (%)	533 (26.1)	281 (23.1)	252 (30.5)	<0.001
PVD, *n* (%)	243 (11.9)	148 (12.2)	95 (11.5)	0.639
Chronic pulmonary disease, *n* (%)	393 (19.2)	217 (17.9)	176 (21.3)	0.053
Diabetes mellitus, *n* (%)	683 (33.4)	413 (34.0)	270 (32.7)	0.536
Malignant cancer, *n* (%)	139 (6.8)	66 (5.4)	73 (8.8)	0.003
Severe liver disease, *n* (%)	35 (1.7)	15 (1.2)	20 (2.4)	0.043
Atrial fibrillation, *n* (%)	773 (37.8)	442 (36.4)	331 (40.0)	0.093
**Laboratory parameters**				
WBC, 10^9^/L	10.4 (7.8–14.3)	8.6 (6.8–10.8)	14.3 (11.2–18.3)	<0.001
Neutrophils percent, %	79.8 (69.2–86.0)	73.0 (64.1–81.6)	85.0 (80.8–89.2)	<0.001
Lymphocytes percent, %	12 (7.2–19.6)	18.0 (12.2–25.2)	6.8 (4.5–9.9)	<0.001
Monocytes percent, %	5.6 (3.8–7.9)	5.5 (3.7–7.8)	5.8 (4.0–8.0)	0.019
Platelets, 10^9^/L	221.0 (169.0–284.0)	217.0 (169.0–277.5)	230.0 (171.0–294.0)	0.013
Hemoglobin, g/dl	12.5 (10.8–13.9)	12.6 (11.0–13.9)	12.2 (10.5–13.9)	0.016
Serum glucose, mg/dl	126.8 (106.7–159.6)	123.0 (103.9–152.9)	134.1 (111.0–169.6)	<0.001
Serum creatinine, mg/dl	1.0 (0.8–1.5)	1.0 (0.8–1.3)	1.1 (0.8–1.7)	<0.001
Serum sodium, mmol/L	141.0 (138.0–143.0)	140.0 (138.0–143.0)	141.0 (138.0–144.0)	0.033
Serum potassium, mmol/L	4.3 (4.0–4.8)	4.3 (4.0–4.7)	4.4 (4.0–4.9)	0.011
Anion gap, mmol/L	16.0 (14.0–19.0)	15.0 (13.0–18.0)	17.0 (15.0–20.0)	<0.001
PT, second	13.2 (11.9–15.5)	13.0 (11.8–14.9)	13.8 (12.2–16.3)	<0.001
PTT, second	30.5 (27.1–39.0)	30.5 (27.1–38.3)	30.6 (27.0–40.6)	0.625
NLR	6.7 (3.6–11.7)	4.1 (2.6–6.6)	12.6 (8.4–19.5)	<0.001
LMR	2.3 (1.4–3.6)	3.2 (2.4–4.8)	1.2 (0.8–1.7)	<0.001
PLR	174.7 (113.7–272.5)	144.1 (99.3–215.0)	238.9 (153.6–377.2)	<0.001
SIRI	3.6 (1.7–7.7)	1.9 (1.2–3.0)	9.1 (6.2–14.9)	<0.001
**Scores**				
SOFA	4 (2–7)	3 (2–6)	5 (3–8)	<0.001
GCS	15 (14–15)	15 (14–15)	15 (14–15)	0.063
**Treatment**				
Thrombolysis, *n* (%)	73 (3.57)	56 (4.6)	17 (2.1)	0.003
Thrombectomy, *n* (%)	35 (1.7)	30 (2.5)	5 (0.6)	0.001
**Clinical Outcomes**				
LOS ICU, day	3.3 (1.7–7.2)	2.9 (1.6–6.6)	4.0 (1.9–8.3)	<0.001
LOS hospital, day	8.8 (4.6–16.9)	7.9 (4.0–15.1)	10.8 (5.7–18.8)	<0.001
Hospital mortality, *n* (%)	392 (19.2)	162 (13.3)	230 (27.8)	<0.001
30-day mortality, *n* (%)	504 (24.7)	215 (17.7)	289 (35.0)	<0.001
90-day mortality, *n* (%)	634 (31.0)	276 (22.7)	358 (43.3)	<0.001
1-year mortality, *n* (%)	779 (38.1)	363 (29.9)	416 (50.3)	<0.001

SIRI, Systemic Inflammation Response Index; CHF, Congestive heart failure; PVD, Peripheral vascular disease; WBC, white blood cell; PT, prothrombin time; APTT, activated partial thromboplastin time; NLR, Neutrophil-to-Lymphocyte Ratio; LMR, Lymphocyte-to-Monocyte Ratio; PLR, Platelet-to-Lymphocyte Ratio; SOFA, Sequential Organ Failure Assessment; GCS, Glasgow coma scale; LOS ICU, Length of ICU stay; LOS hospital, Length of hospital stay.

As shown in Table 1, patients with high SIRI values were more likely to have higher WBC count, neutrophil percentage, monocyte percentage, platelet count, serum glucose, serum creatinine, serum sodium, serum potassium, anion gap, PT, NLR, PLR, and SOFA score. However, they had lower lymphocyte percentage, hemoglobin, LMR, thrombolysis percent, and thrombectomy percent. Furthermore, patients with high SIRI values tended to have a history of CHF, malignant cancer, and severe liver disease, while tending not to have dyslipidemia.

### Relationship between the SIRI and the clinical outcomes before PSM

Compared with patients in the SIRI <4.57 group, patients with SIRI ≥4.57 were at a higher risk of prolonged ICU stay (4.0 vs. 2.9 days, *p* < 0.001), prolonged hospital stay (10.8 vs. 7.9 days, *p* < 0.001), hospital mortality (27.8 vs. 13.3%, *p* < 0.001), 30-day mortality (35.0 vs. 17.7%, *p* < 0.001), 90-day mortality (43.3 vs. 22.7%, *p* < 0.001), and 1-year mortality (50.3 vs. 29.9%, *p* < 0.001) (Table 1).

**Figure 2:**
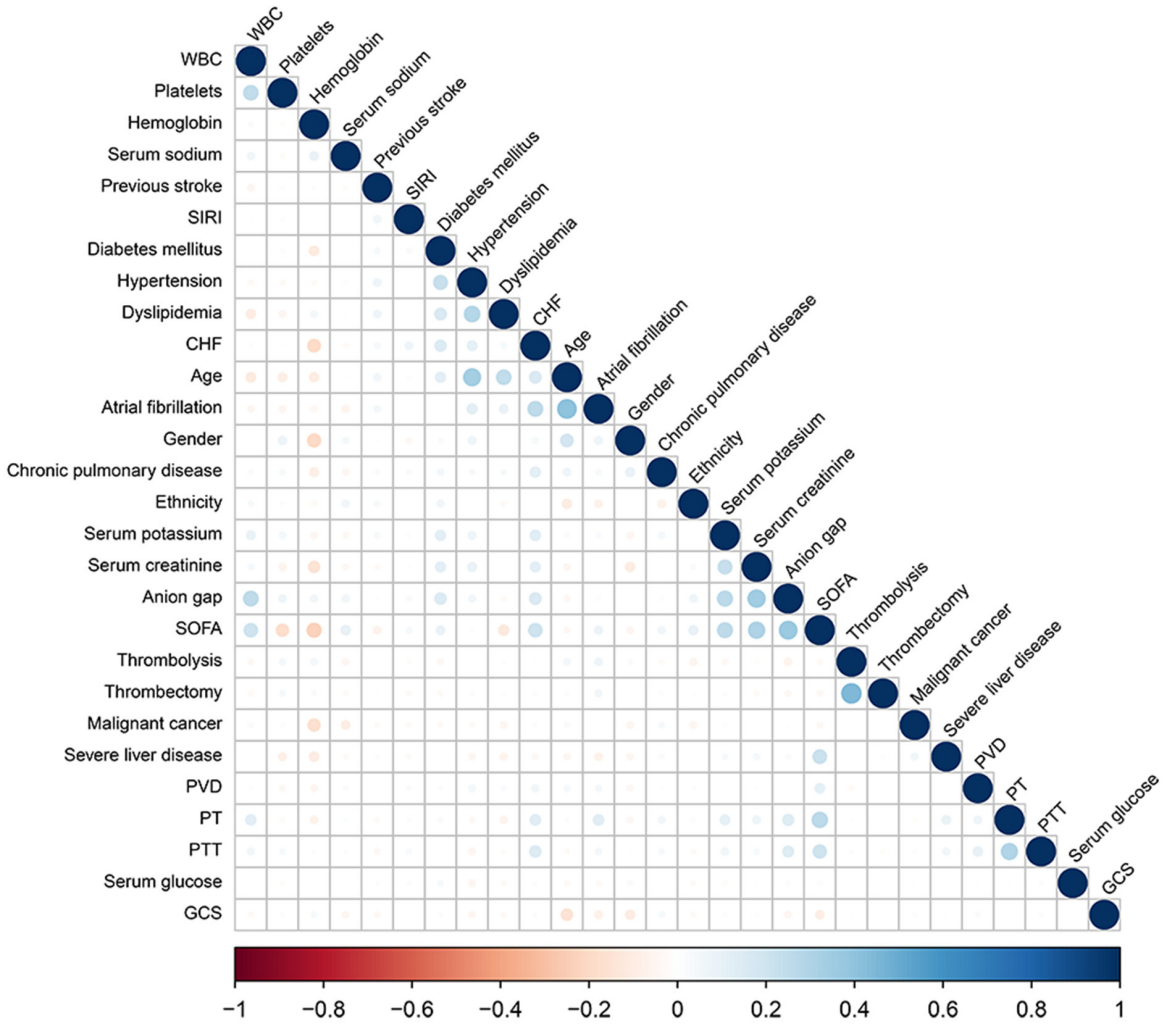
Correlation coefficient between the variables. A color scale represents positive correlation (in blue) to negative correlation (in red). The size of the circle represents the absolute values of correlation coefficients.

To verify the independent relationship between SIRI and the mortality of patients with AIS, we performed univariate and multivariate Cox regression analyses (Table 2), with SIRI stratified by binary and quartile. Before we performed the Cox regression analysis, the Pearson correlation coefficient was used to ensure that there was no collinearity existed between variables. The results of correlation analyses are presented in Figure 2.

**Table 2:** Univariate and multivariate Cox regression models used to study the association of SIRI with mortality in patients with AIS.

**Clinical outcome**	**Non-adjusted**		**Model I**		**Model II**	
	**HR (95% CI)**	***p*** **-value**	**HR (95% CI)**	***p*** **-value**	**HR (95% CI)**	***p*** **-value**
**90-day all-cause mortality**						
**Before PSM**						
SIRI (≥4.57)	2.20 (1.88–2.58)	<0.001	2.18 (1.86–2.55)	<0.001	1.56 (1.30–1.89)	<0.001
SIRI (quartile)						
<2.59	1		1		1	
2.59–4.57	1.02 (0.78–1.32)	0.895	1.02 (0.79–1.33)	0.859	1.00 (0.77–1.31)	0.994
4.57–7.30	1.70 (1.34–2.16)	<0.001	1.71 (1.34–2.17)	<0.001	1.41 (1.10–1.81)	0.008
>7.30	2.53 (2.02–3.17)	<0.001	2.54 (2.02–3.18)	<0.001	1.66 (1.26–2.19)	<0.001
*p* for trend		<0.001		<0.001		<0.001
**After PSM**						
SIRI (≥4.57)	1.46 (1.16–1.84)	0.001	1.50 (1.19–1.89)	0.001	1.47 (1.16–1.86)	0.001
**30-day all-cause mortality**						
**Before PSM**						
SIRI (≥4.57)	2.21 (1.85–2.64)	<0.001	2.16 (1.81–2.58)	<0.001	1.54 (1.25–1.90)	<0.001
SIRI (quartile)						
<2.59	1		1		1	
2.59–4.57	0.96 (0.71–1.29)	0.772	0.95 (0.71–1.28)	0.760	0.94 (0.69–1.27)	0.683
4.57–7.30	1.66 (1.27–2.17)	<0.001	1.63 (1.25–2.13)	<0.001	1.35 (1.02–1.79)	0.036
>7.30	2.47 (1.92–3.18)	<0.001	2.43 (1.88–3.13)	<0.001	1.58 (1.16–2.14)	0.004
*p* for trend		<0.001		<0.001		0.002
**After PSM**						
SIRI (≥4.57)	1.42 (1.10–1.84)	0.007	1.46 (1.12–1.89)	0.004	1.41 (1.08–1.84)	0.011
**1-year all-cause mortality**						
**Before PSM**						
SIRI (≥4.57)	2.01 (1.75–2.32)	<0.001	2.01 (1.75–2.32)	<0.001	1.44 (1.23–1.70)	<0.001
SIRI (quartile)						
<2.59	1		1		1	
2.59–4.57	0.96 (0.76–1.21)	0.717	0.97 (0.77–1.22)	0.781	0.95 (0.75–1.20)	0.647
4.57–7.30	1.60 (1.29–1.97)	<0.001	1.62 (1.31–1.99)	<0.001	1.37 (1.09–1.71)	0.006
>7.30	2.27 (1.86–2.77)	<0.001	2.30 (1.88–2.82)	<0.001	1.58 (1.23–2.02)	<0.001
*p* for trend		<0.001		<0.001		<0.001
**After PSM**						
SIRI (≥4.57)	1.35 (1.10–1.66)	0.004	1.40 (1.14–1.72)	0.001	1.41 (1.15–1.74)	0.001

Non-adjusted model was adjusted for none.

Model I was adjusted for age, gender, and ethnicity.

Model II was adjusted for age, gender, ethnicity, hypertension, congestive heart failure, chronic pulmonary disease, diabetes mellitus, malignant cancer, severe liver disease, atrial fibrillation, white blood cell, hemoglobin, serum creatinine, serum sodium, serum potassium, anion gap, prothrombin time, activated partial thromboplastin time, thrombolysis, and thrombectomy.

SIRI, Systemic Inflammation Response Index; HR, hazard ratio; CI, confidence interval; PSM, propensity score matching; AIS, acute ischemic stroke.

In the non-adjusted model, a high level of SIRI (≥4.57) was associated with an increased risk of 90-day (HR = 2.20, 95% CI: 1.88–2.58, *p* < 0.001), 30-day (HR = 2.21, 95% CI: 1.85–2.64, *p* < 0.001), and 1-year (HR = 2.01, 95% CI: 1.75–2.32, *p* < 0.001) all-cause mortality. In the multivariate model I, after adjusting for age, gender, and ethnicity, the SIRI ≥4.57 group also showed a higher risk of 90-day (HR = 2.18, 95% CI: 1.86–2.55, *p* < 0.001), 30-day (HR = 2.16, 95% CI: 1.81–2.58, *p* < 0.001), and 1-year (HR = 2.01, 95% CI: 1.75–2.32, *p* < 0.001) all-cause mortality. In model II, after adjusting for the variables in the model I and other possible confounders, a high SIRI level was still associated with an increased risk of 90-day (HR = 1.54, 95% CI: 1.29–1.84, *p* < 0.001), 30-day (HR = 1.51, 95% CI: 1.23–1.84, *p* < 0.001), and 1-year (HR = 1.44, 95% CI: 1.23–1.70, *p* < 0.001) all-cause mortality. A similar trend in these outcomes was also found using the quartile of the distribution of SIRI level (Table 2). This indicated that higher SIRI, in the third and fourth SIRI quartile, was associated with increased risk of 90-day, 30-day, and 1-year all-cause mortality in patients with AIS, without any significant difference between the first and second quartile of patients in lower SIRI level (Table 2).

**Figure 3:**
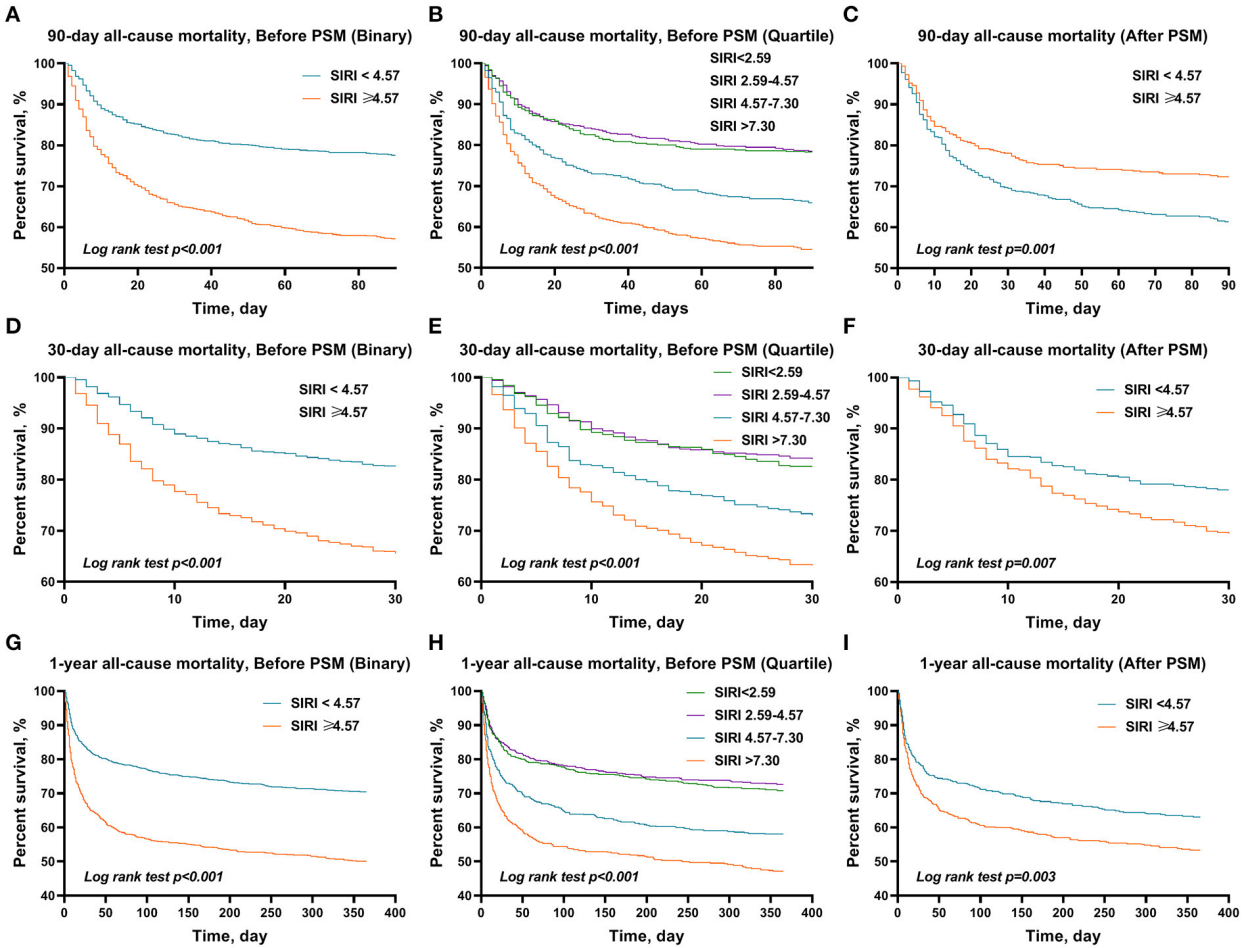
The Kaplan–Meier survival plots of overall survival. A significantly lower 90-day **(A)**, 30-day **(B)**, and 1-year **(C)** survival rate was observed among patients in the higher SIRI (≥4.57) before PSM. It also showed significantly lower 90-day **(D)**, 30-day **(E)**, and 1-year **(F)** survival rates in the third and fourth SIRI quartiles compared with the first quartile in patients with AIS. After PSM, a similar trend in these outcomes was also found in 90-day **(G)**, 30-day **(H)**, and 1-year **(I)** overall survival. *p*-value was calculated by log-rank test and indicated in the plot. SIRI, systemic inflammation response index; PSM, propensity score matching.

The Kaplan–Meier survival curves comparing the two groups are shown in Figure 2. Patients with SIRI ≥4.57 had a significantly lower 90-day, 30-day, and 1-year survival rate compared to patients with SIRI <4.57 (56.7 vs. 77.3%, *p* < 0.001; 65.0 vs. 86.7%, *p* < 0.001; 49.7 vs. 70.1%, *p* < 0.001). Similarly, among the distribution quartiles of the SIRI level, Kaplan–Meier curves also showed significantly lower 90-day, 30-day, and 1-year survival rates in the third and fourth SIRI quartiles compared with the lower SIRI in the first and second quartiles (*p* < 0.001 for all) (Figure 3).

### Sensitivity analysis

**Figure 4:**
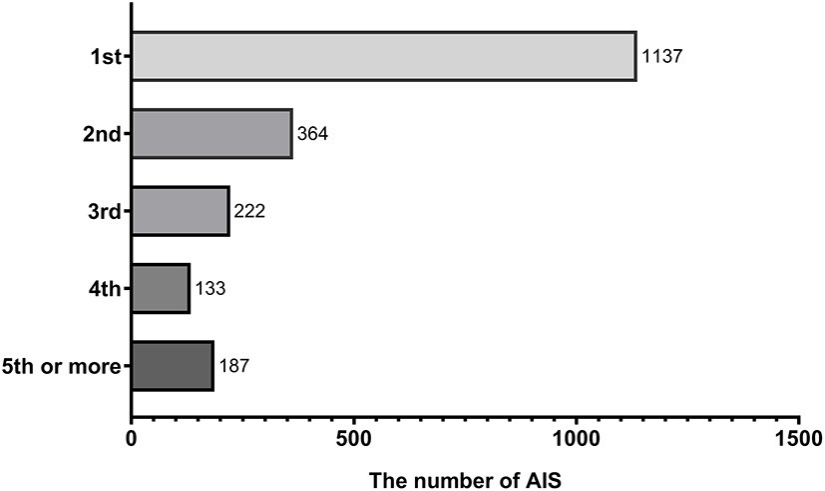
The number of acute ischemic strokes in a different sequence of diagnosis.

As we recruited all sequences of diagnosis of patients with AIS, sensitivity analysis was conducted based on the first or non-first diagnosis of AIS to identify our outcomes. The sequence of diagnosis of patients with AIS is shown in Figure 4. To further test the robustness of our results, we performed a sensitivity analysis. In the first diagnosis of patients with AIS, a high level of SIRI (≥4.57) was associated with an increased risk of 90-day (HR =1.47, 95% CI: 1.11–1.96, *p* = 0.008), 30-day (HR = 1.55, 95% CI: 1.14–2.10, *p* =0.005), and 1-year (HR = 1.33, 95% CI: 1.03–1.72, *p* = 0.031) all-cause mortality. The association between SIRI and the risk of 90-day, 30-day, and 1-year all-cause mortality remained stable in those non-first sequence of diagnosis of patients with AIS (HR = 1.57, 95% CI: 1.21–2.05, *p* = 0.001; HR = 1.42, 95% CI: 1.05–1.93, *p* = 0.025; and HR = 1.55, 95% CI: 1.22–1.97, *p* < 0.001, respectively) (Table 3).

**Table 3:** Sensitivity analysis of the association between SIRI and all-cause mortality in AIS patients (adjustment through multivariate Cox regression models).

	**Case, *n* (%)**	**Univariate**		**Multivariate**	
		**HR (95% CI)**	***p*** **-value**	**HR (95% CI)**	***p*** **-value**
**90-day all cause mortality**					
**The first sequence of diagnosis of AIS**					
SIRI (≥4.57)	370 (32.5)	2.26 (1.81–2.83)	<0.001	1.47 (1.11–1.96)	0.008
**Non-first sequence of diagnosis of AIS**					
SIRI (≥4.57)	457 (50.4)	1.99 (1.59–2.49)	<0.001	1.57 (1.21–2.05)	0.001
**30-day all cause mortality**					
**The first sequence of diagnosis of AIS**					
SIRI (≥4.57)	370 (32.5)	2.52 (1.97–3.22)	<0.001	1.55 (1.14–2.10)	0.005
**Non-first sequence of diagnosis of AIS**					
SIRI (≥4.57)	457 (50.4)	1.87 (1.44–2.42)	<0.001	1.42 (1.05–1.93)	0.025
**1-year all cause mortality**					
**The first sequence of diagnosis of AIS**					
SIRI (≥4.57)	370 (32.5)	1.99 (1.63–2.45)	<0.001	1.33 (1.03–1.72)	0.031
**Non-first sequence of diagnosis of AIS**					
SIRI (≥4.57)	457 (50.4)	1.86 (1.52–2.27)	<0.001	1.55 (1.22–1.97)	<0.001

Non-adjusted model was adjusted for none.

Adjusted model was adjusted for age, gender, ethnicity, hypertension, congestive heart failure, chronic pulmonary disease, diabetes mellitus, malignant cancer, severe liver disease, atrial fibrillation, white blood cell, hemoglobin, serum creatinine, serum sodium, serum potassium, anion gap, prothrombin time, activated partial thromboplastin time, thrombolysis, and thrombectomy.

SIRI, Systemic Inflammation Response Index; HR, hazard ratio; CI, confidence interval; AIS, acute ischemic stroke.

### Relationship between the SIRI and clinical outcomes after PSM

**Figure 5:**
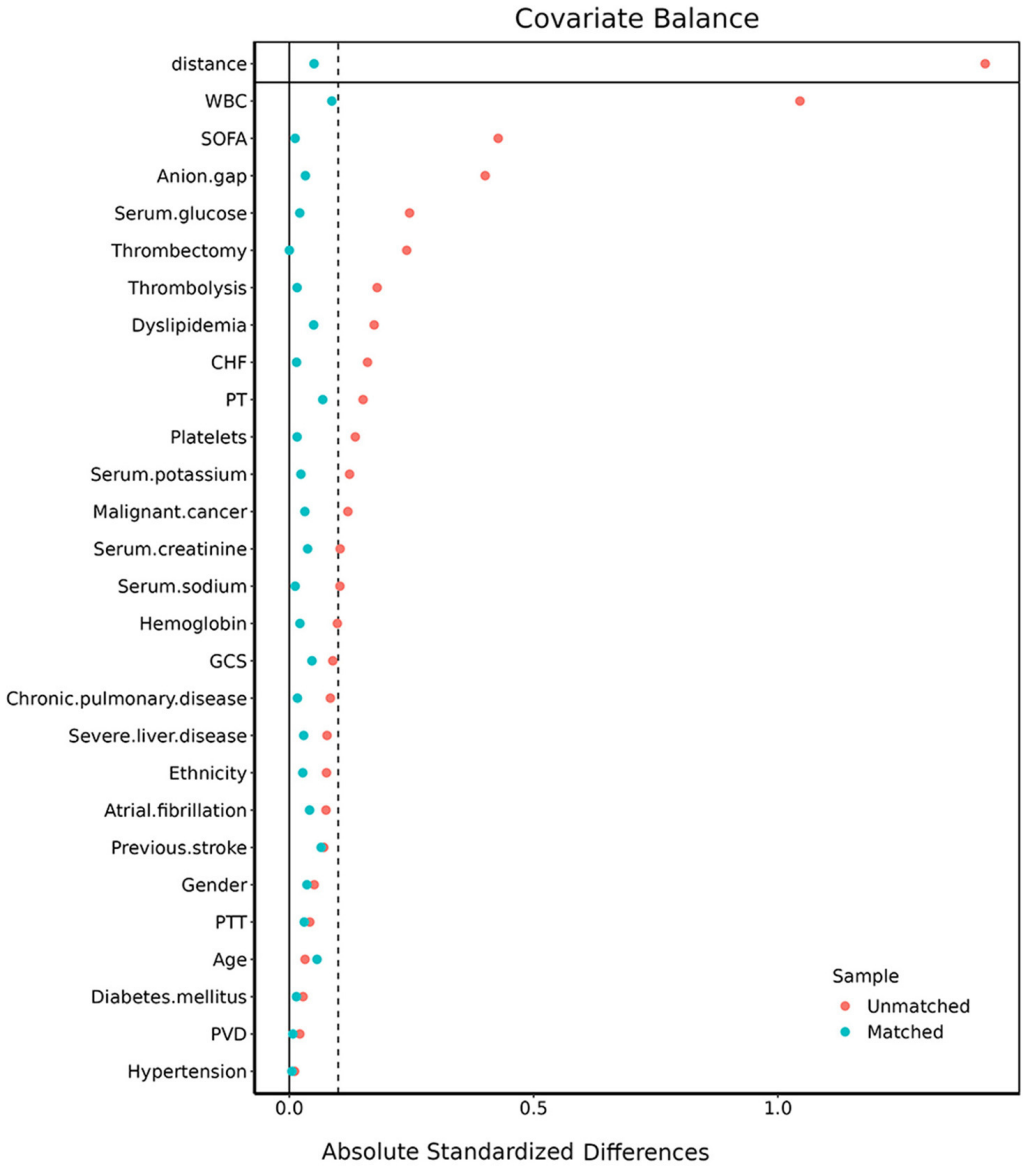
The absolute standardized differences for variables used to match the two groups.

Considering the imbalanced baseline characteristics between patients in the low (<4.57) SIRI group and high (≥4.57) SIRI group, we performed a 1:1 ratio PSM analysis to balance the covariate variables, and 444 pairs of score-matched patients were generated. The baseline characteristics of patients after PSM are shown in Table 4. The demographics, comorbidities, most laboratory parameters, metrics, and treatment were well-balanced between the two groups. Because the lymphocyte percentage, neutrophil percentage, monocyte percentage, NLR, LMR, and PLR directly influenced the SIRI value, we did not include them in the matched variables. Absolute standardized differences (ASD) were calculated before and after PSM to assess the quality of matching (Figure 5).

**Table 4:** Demographic and clinical characteristics after propensity score matching.

**Characteristic**	**Total (*n* = 888)**	**SIRI**		***p-*value**
		<**4.57 (*****n*** = **444)**	≥**4.57 (*****n*** = **444)**	
**Demographics**				
Age, years	71.5 (58.6–82.8)	71.8 (58.2–84.2)	71.1 (58.8–81.6)	0.245
Male, *n* (%)	454 (51.1)	231 (52)	223 (50.2)	0.638
Ethnicity, *n* (%)				0.916
White	546 (61.5)	276 (62.2)	270 (60.8)	
Black	91 (10.2)	45 (10.1)	46 (10.4)	
Others	251 (28.3)	123 (27.7)	128 (28.8)	
**Comorbidities**				
Hypertension, *n* (%)	685 (77.1)	342 (77)	343 (77.3)	1.000
Dyslipidemia, *n* (%)	445 (50.1)	217 (48.9)	228 (51.4)	0.502
Previous stroke, *n* (%)	95 (10.7)	43 (9.7)	52 (11.7)	0.385
CHF, *n* (%)	253 (28.5)	128 (28.8)	125 (28.2)	0.882
PVD, *n* (%)	103 (11.6)	51 (11.5)	52 (11.7)	1.000
Chronic pulmonary disease, *n* (%)	185 (20.8)	94 (21.2)	91 (20.5)	0.869
Diabetes mellitus, *n* (%)	295 (33.2)	146 (32.9)	149 (33.6)	0.887
Malignant cancer, *n* (%)	58 (6.5)	31 (7)	27 (6.1)	0.684
Severe liver disease, *n* (%)	14 (1.6)	6 (1.4)	8 (1.8)	0.788
Atrial fibrillation, *n* (%)	371 (41.8)	190 (42.8)	181 (40.8)	0.586
**Laboratory parameters**				
WBC, 109/L	11.6 (9.6–13.8)	11.6 (9.3–13.7)	11.8 (9.7–13.9)	0.169
Neutrophils percent, %	81.7 (75.0–86.8)	77.9 (69.8–84.0)	84.0 (79.6–88.5)	<0.001
Lymphocytes percent, %	10.4 (6.8–15.6)	15.0 (10.8–20.4)	7.5 (5.0–10.1)	<0.001
Monocytes percent, %	5.2 (3.5–7.6)	4.2 (2.9–6.2)	6.2 (4.3–8.6)	<0.001
Platelets, 109/L	223.5 (168.5–288.5)	223.0 (173.0–291.0)	224.0 (167.0–285.0)	0.612
Hemoglobin, g/dl	12.4 (10.6–13.9)	12.4 (10.6–13.8)	12.3 (10.7–14.0)	0.764
Serum glucose, mg/dl	128.8 (107.7–160.8)	128.1 (109.3–160.3)	129.0 (107.2–161.2)	0.863
Serum creatinine, mg/dl	1.1 (0.8–1.6)	1.1 (0.8–1.6)	1.1 (0.8–1.6)	0.685
Serum sodium, mmol/L	141.0 (138.0–143.0)	141.0 (138.0–143.0)	140.0 (138.0–143.0)	0.636
Serum potassium, mmol/L	4.3 (4.0–4.8)	4.3 (4.0–4.8)	4.3 (4.0–4.9)	0.98
Anion gap, mmol/L	16.0 (14.0–19.0)	16.0 (14.0–19.0)	16.0 (14.0–19.0)	0.358
PT, second	13.4 (12.0–15.5)	13.5 (12.0–15.7)	13.4 (12.1–15.4)	0.653
PTT, second	30.1 (26.6–36.7)	30.2 (26.8–36.6)	29.9 (26.4–36.9)	0.501
NLR	7.8 (4.8–12.7)	5.1 (3.4–7.8)	11.2 (7.8–17.6)	<0.001
LMR	2.0 (1.2–3.4)	3.4 (2.6–4.9)	1.2 (0.9–1.6)	<0.001
PLR	181.2 (117.2–300.9)	132.3 (85.7–199.3)	253.9 (170.7–412.5)	<0.001
SIRI	4.6 (2.6–7.4)	2.6 (1.7–3.6)	7.4 (5.7–11.2)	<0.001
**Scores**				
SOFA	4.0 (2.5–7.0)	4.0 (2.0–7.0)	4.0 (3.0–7.0)	0.942
GCS	15.0 (14.0–15.0)	15.0 (14.0–15.0)	15.0 (14.0–15.0)	0.894
**Treatment**				
Thrombolysis, *n* (%)	23 (2.6)	11 (2.5)	12 (2.7)	1.000
Thrombectomy, *n* (%)	8 (0.9)	4 (0.9)	4 (0.9)	1.000
**Clinical Outcomes**				
LOS ICU, day	3.7 (1.8–7.4)	3.5 (1.7–7.3)	3.9 (1.9–7.5)	0.269
LOS hospital, day	9.7 (5.0–17.7)	9.1 (4.9–17.6)	9.9 (5.4–17.8)	0.446
Hospital mortality, *n* (%)	172 (19.4)	75 (16.9)	97 (21.8)	0.075
30-day mortality, *n* (%)	237 (26.7)	100 (22.5)	137 (30.9)	0.006
90-day mortality, *n* (%)	298 (33.6)	125 (28.2)	173 (39)	<0.001
1-year mortality, *n* (%)	374 (42.1)	166 (37.4)	208 (46.8)	0.005

SIRI, Systemic Inflammation Response Index; CHF, Congestive heart failure; PVD, Peripheral vascular disease; WBC, white blood cell; PT, prothrombin time; APTT, activated partial thromboplastin time; NLR, Neutrophil-to-Lymphocyte Ratio; LMR, Lymphocyte-to-Monocyte Ratio; PLR, Platelet-to-Lymphocyte Ratio; SOFA, Sequential Organ Failure Assessment; GCS, Glasgow coma scale; LOS ICU, Length of ICU stay; LOS hospital, Length of hospital stay.

After PSM, significant differences between the two groups were still observed in 90-day (39.0 vs. 28.2%, *p* < 0.001), 30-day (30.9 vs. 22.5%, *p* = 0.006), and 1-year (46.8 vs. 37.4%, *p* = 0.005) all-cause mortality, but not in ICU stay (3.9 vs. 3.5 days, *p* = 0.269), hospital stay (9.9 vs. 9.1 days, *p* = 0.446), and hospital mortality (21.8 vs. 16.9%, *p* = 0.075) (Table 4). Similarly, the results of the multivariate Cox regression analyses in patients after PSM indicated that SIRI ≥4.57 remained an independent predictor of higher 90-day (HR = 1.47, 95% CI: 1.16–1.86, *p* = 0.001), 30-day (HR = 1.41, 95% CI: 1.08–1.84, *p* = 0.011), and 1-year (HR = 1.41, 95% CI: 1.15–1.74, *p* = 0.001) all-cause mortality (Table 2). Additionally, the Kaplan–Meier survival curves comparing the two groups showed that after PSM, patients with SIRI ≥4.57 still had a significantly lower 90-day (61.0 vs. 71.8%, *p* = 0.001), 30-day (69.1 vs. 77.5%, *p* = 0.007), and 1-year (53.2 vs. 62.6%, *p* = 0.003) survival rate compared to patients with SIRI <4.57 (Figure 2).

### Subgroup analysis for the effect of SIRI on clinical outcomes in patients with AIS

**Figure 6:**
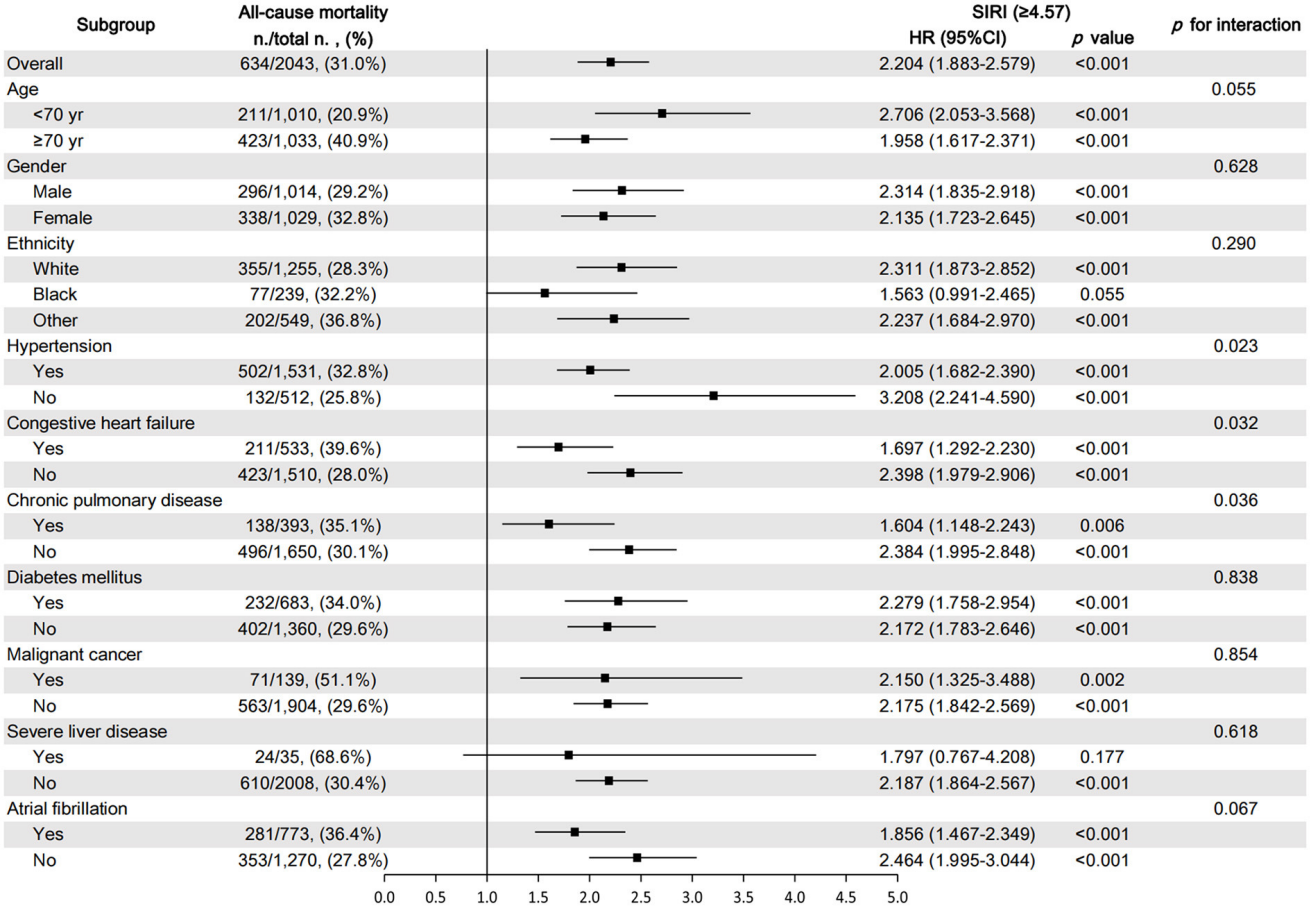
Subgroup analysis for the effect of SIRI on 90-day all-cause mortality in critically ill patients with AIS.

The subgroup analysis was performed to reveal the correlation between SIRI and 90-day all-cause mortality according to age (<70 and ≥70 years), gender, ethnicity, and comorbidities. The results showed that in all subgroups, the increase in SIRI level was closely related to the increase in the 90-day all-cause mortality of critically ill patients with AIS (Figure 6). Most of the stratification factors were not found to have a significant impact on the relationship between SIRI and 90-day all-cause mortality, except for hypertension (*p* = 0.023), CHF (*p* = 0.032), and chronic pulmonary disease (*p* = 0.036).

### Prognostic value of the SIRI and other parameters

**Figure 7:**
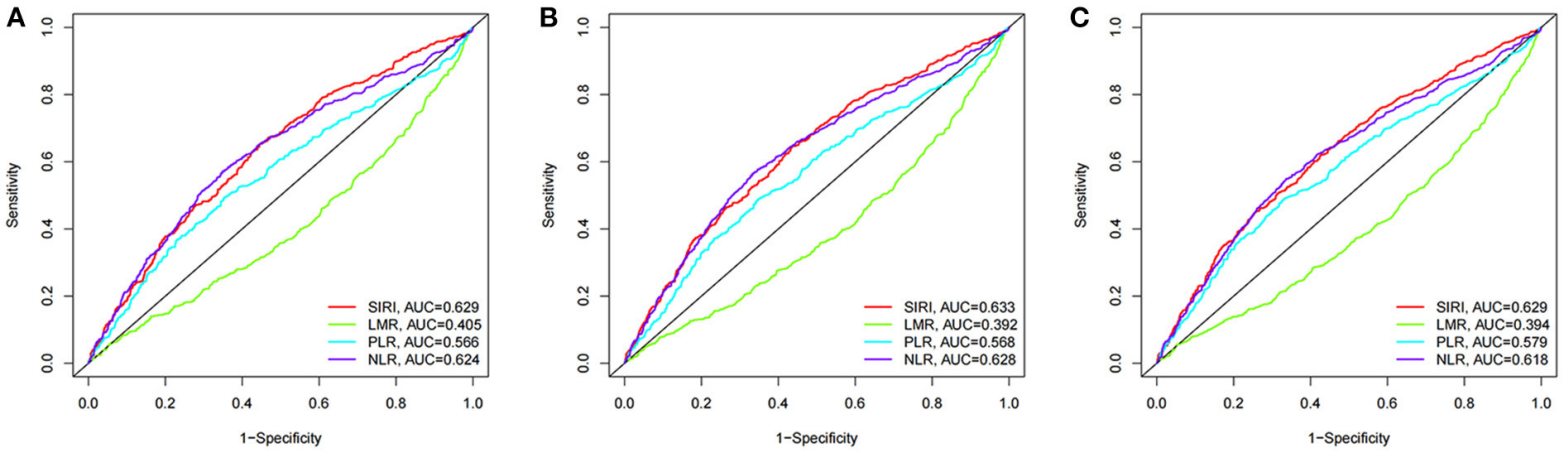
The time-dependent ROC analysis for prognostic value in patients with acute ischemic stroke. **(A)** ROC curves corresponding to 90-day all-cause mortality. **(B)** ROC curves corresponding to 30-day all-cause mortality. **(C)** ROC curves corresponding to 1-year all-cause mortality.

We compared the prognostic efficiency of the SIRI and NLR, LMR, and PLR in patients with AIS by the time-dependent receiver operating characteristic (ROC) analysis (Figure 7). It was revealed that the performance of SIRI was better than LMR and PLR in predicting 90-day (AUC 0.629 vs. 0.405; 0.629 vs. 0.566), 30-day (AUC 0.633 vs. 0.392; 0.633 vs. 0.568), and 1-year (AUC 0.629 vs. 0.394; 0.629 vs. 0.579) all-cause mortality in patients with AIS. However, it showed a similar predictive value compared with NLR in 90-day, 30-day, and 1-year all-cause mortality in patients with AIS (AUC 0.629 vs. 0.624; 0.633 vs. 0.628; 0.629 vs. 0.618, respectively).

## Discussion

In this study, we investigated the association between SIRI and the risk of 90-day, 30-day, and 1-year all-cause mortality in patients with AIS in a cohort study. A PSM analysis was performed to balance the potential confounding factors. It was found that a higher SIRI could be more likely to be associated with poor outcomes in patients with AIS. Thus, the higher the SIRI level, the higher the risk of 90-day, 30-day, and 1-year mortality. The correlation remained significant after adjusting for possible confounders, stratifying according to comorbidities, and performing PSM, respectively.

Several studies have demonstrated the relevance of inflammation in the pathogenesis of ischemic stroke. First, inflammation is involved in all stages of the atherosclerotic plaque, leading to thrombotic events (19). Monocyte adherence to the vascular endothelium, migration into the arterial intima, and subsequent differentiation into foamy macrophages are the key events in early plaque initiation (20, 21). Disruption of atherosclerotic plaques, which often leads to acute ischemic stroke, is associated with monocyte/macrophage and T-cell infiltration (22). Second, inflammation plays an essential role in the pathophysiological processes of cerebral ischemic injury (5). After ischemia, circulating white cells extravasate into the brain and meninges. Neutrophils damage the brain by releasing proteases including metalloproteases (MMP-9), cathepsin G, reactive oxygen and nitrogen species, and inflammatory IL-1β (23). Monocyte-derived macrophage (MDM) influx into the ischemic brain may be important for regulating the immune response after stroke (24, 25). The third and most important factor is that acute ischemic stroke activates systemic inflammation and neurohumoral pathways, which could trigger or aggravate immune activation, immunodepression, and functional impairment of multiple peripheral organs (5, 25–29). Thus, inflammatory parameters may be associated with the outcome after AIS.

CRP concentration was first found to be an independent predictor of survival after ischemic stroke (30). Subsequently, studies have shown that various inflammatory parameters, such as activated leukocyte cell adhesion molecule (31), serum chemokines-12 (32), high-sensitivity C-reactive protein (33), E-selectin (34), Interleukin-6 (35), monocyte chemoattractant protein (36), and others, were associated with the risk of poor outcome after stroke (37). Due to the limitation of a single indicator, new biomarkers combining multiple indicators into a new predictor, such as neutrophil-lymphocyte ratio (NLR), lymphocyte-to-monocyte (LMR), and platelet-lymphocyte ratio (PLR), have been designed to investigate outcomes after AIS. Studies have shown that both NLR and PLR were independently associated with poor short-term outcomes of patients with AIS (8, 38), and low LMR was associated with worse outcomes at 3 months after stroke onset (39).

The SIRI, which combines three inflammatory indicators, is a comprehensive, easily accessible, and inexpensive marker of chronic low-grade inflammation. Moreover, it was found to be an independent prognostic indicator in cancer (14), rheumatoid arthritis (15), mechanical thrombectomy for large artery occlusion (16), and intracerebral hemorrhage (17). In our study, high SIRI levels tended to be associated with 90-day, 30-day, and 1-year adverse outcomes. There was no significant difference between the 1st and 2nd quartile, and the 4th quartile had a 2.2-fold (90-day), 2.21-fold (30-day), and 2.01-fold (1-year) higher risk of mortality compared to the 1st quartile. Therefore, we consider that higher SIRI was associated with a higher risk of mortality. In addition, the subgroup analysis results demonstrated that the association between high SIRI levels and poor 90-day mortality was stable and consistent across AIS patients with different comorbidities. We also noted that patients complicated with hypertension, CHF, and chronic pulmonary disease had a higher risk of 90-day mortality, and this risk was higher for patients with lower SIRI, which implied that SIRI may be more valuable for the prognostic evaluation of AIS patients without hypertension, CHF, or chronic pulmonary disease.

A similar association between SIRI and AIS has also been observed in recent studies (18, 40, 41). However, patients with high SIRI had a trend of increased comorbidities and higher scores for SOFA. High scores of SOFA are independently associated with high mortality. To guarantee the robustness of the findings, we used PSM to reduce the baseline differences between the two groups. After balancing the difference of score and comorbidities between the two groups by PSM analysis, patients with a high SIRI (≥4.57) still had a 1.47-fold (90-day), 1.41-fold (30-day), and 1.41-fold (1-year) higher risk of mortality compared to the low SIRI (<4.57) patients. Therefore, it is suggested that high SIRI is an independent risk factor of mortality in patients with AIS.

Although a high SIRI level was helpful in the discrimination of patients at risk of poor outcomes, the predictive performance of SIRI is only satisfactory for 90-day, 30-day, and 1-year mortality (AUC was 0.629, 0.633, and 0.629). Consequently, using a single measurement of SIRI may not be an effective tool for assessing risk following an ischemic stroke. Further evaluation strategy should be considered to prove the clinical value of SIRI among patients with acute ischemic stroke.

The main strength of our study is that it was based on large, real-world data, and we created comparable groups through group matching, thereby attempting to reduce bias due to confounding variables. There were still several limitations in this study. First, we excluded patients who had some missing important variables, such as WBC subtypes, which could have resulted in selection bias. Second, it was a single-center study, and the prognostic value of SIRI for AIS should be further confirmed in different populations and countries. Third, because of the retrospective collection of the data, variables were not evenly distributed across groups, although PSM analysis was conducted to minimize differences between the groups. Fourth, because the ICD code is a final diagnosis, super-acute complications such as stunned heart syndrome, pneumonia, or sepsis were not included in our study, which may cause a high level of SIRS index due to strongly exacerbating cerebral perfusion and tissue necrosis. Finally, in our study, SIRI only presented a satisfactory prediction ability for 90-day, 30-day, and 1-year mortality.

## Conclusion

High SIRI level was correlated with poor clinical outcomes in critically ill patients with AIS and was an independent risk factor for 30-day, 90-day, and 1-year all-cause mortality. However, to confirm the role of SIRI as a predictor for the prognosis of patients with ischemic stroke, further prospective case–control studies are required.

## Data availability statement

The data analyzed in this study was obtained from the Medical Information Mart for Intensive Care IV (MIMIC-IV) Database, the following licenses/restrictions apply: To access the files, users must be a credentialed user, complete the required training (CITI Data or Specimens Only Research) and sign the data use agreement for the project. Requests to access these datasets should be directed to PhysioNet, https://physionet.org/, doi: 10.13026/a3wn-hq05.

## Ethics statement

The studies involving human participants were reviewed and approved by Institutional Review Boards of Massachusetts Institute of Technology (Cambridge, MA, USA) and Beth Israel Deaconess Medical Center (Boston, MA, USA). Written informed consent for participation was not required for this study in accordance with the national legislation and the institutional requirements.

## Author contributions

HL designed the study. HD drafted the manuscript. HD and ML collected and analyzed the clinical data. WM, LC, and SW participated in the implementation of statistical methods in this study and put forward constructive suggestions. JS and XM reviewed the study and participated in the interpretation of the results. All authors gave final approval of the version to be published and agree to be accountable for all aspects of the work.

## References

[1] GBD 2019 Stroke Collaborators. Global, regional, and national burden of stroke and its risk factors, 1990-2019: a systematic analysis for the Global Burden of Disease Study 2019. Lancet Neurol. (2021) 20:795–820. 10.1016/S1474-4422(21)00252-0 34487721 PMC8443449

[2] Randolph SA. Ischemic stroke. Workplace Health Saf. (2016) 64:444. 10.1177/2165079916665400 27621261

[3] Anrather J Iadecola C. Inflammation and stroke: an overview. Neurotherapeutics. (2016) 13:661–70. 10.1007/s13311-016-0483-x 27730544 PMC5081118

[4] Chamorro Á Dirnagl U Urra X Planas AM. Neuroprotection in acute stroke: targeting excitotoxicity, oxidative and nitrosative stress, and inflammation. Lancet Neurol. (2016) 15:869–81. 10.1016/S1474-4422(16)00114-9 27180033

[5] Iadecola C Buckwalter MS Anrather J. Immune responses to stroke: mechanisms, modulation, and therapeutic potential. J Clin Invest. (2020) 130:2777–88. 10.1172/JCI135530 32391806 PMC7260029

[6] Cui P McCullough LD Hao J. Brain to periphery in acute ischemic stroke: mechanisms and clinical significance. Front Neuroendocrinol. (2021) 63:100932. 10.1016/j.yfrne.2021.100932 34273406 PMC9850260

[7] Ferro D Matias M Neto J Dias R Moreira G Petersen N. Neutrophil-to-lymphocyte ratio predicts cerebral edema and clinical worsening early after reperfusion therapy in stroke. Stroke. (2021) 52:859–67. 10.1161/STROKEAHA.120.032130 33517702

[8] Li W Hou M Ding Z Liu X Shao Y Li X. Prognostic value of neutrophil-to-lymphocyte ratio in stroke: a systematic review and meta-analysis. Front Neurol. (2021) 12:686983. 10.3389/fneur.2021.686983 34630275 PMC8497704

[9] Yan Y-K Huang H Li D-P Ai Z-Y Li X Sun Z. Prognostic value of the platelet-to-lymphocyte ratio for outcomes of stroke: a systematic review and meta-analysis. Eur Rev Med Pharmacol Sci. (2021) 25:6529–6538. 10.26355/eurrev_202111_27095 34787855

[10] Huang Y-H Wang Z-T Zhao B Ma Y-H Ou Y-N Hu H. An elevated platelet-to-lymphocyte ratio is associated with a higher risk of intracranial atherosclerotic stenosis. Chin Med J. (2022) 135:1425–31. 10.1097/CM9.0000000000002228 35868006 PMC9481432

[11] Li Q Ma X Shao Q Yang Z Wang Y Gao F. Prognostic impact of multiple lymphocyte-based inflammatory indices in acute coronary syndrome patients. Front Cardiovasc Med. (2022) 9:811790. 10.3389/fcvm.2022.811790 35592392 PMC9110784

[12] Huang L Peng J Wang X Li F. High platelet-lymphocyte ratio is a risk factor for 30-day mortality in in-hospital cardiac arrest patients: a case-control study. Expert Rev Clin Immunol. (2021) 17:1231–9. 10.1080/1744666X.2021.1994389 34696670

[13] Chen Y Wang W Zeng L Mi K Li N Shi J. Association between neutrophil-lymphocyte ratio and all-cause mortality and cause-specific mortality in US adults, 1999-2014. Int J Gen Med. (2021) 14:10203–11. 10.2147/IJGM.S339378 34992439 PMC8710673

[14] Chao B Ju X Zhang L Xu X Zhao Y. A novel prognostic marker systemic inflammation response index (SIRI) for operable cervical cancer patients. Front Oncol. (2020) 10:766. 10.3389/fonc.2020.00766 32477958 PMC7237698

[15] Xu Y He H Zang Y Yu Z Hu H Cui J. Systemic inflammation response index (SIRI) as a novel biomarker in patients with rheumatoid arthritis: a multi-center retrospective study. Clin Rheumatol. (2022) 41:1989–2000. 10.1007/s10067-022-06122-1 35266094

[16] Yi HJ Sung JH Lee DH. Systemic inflammation response index and systemic immune-inflammation index are associated with clinical outcomes in patients treated with mechanical thrombectomy for large artery occlusion. World Neurosurg. (2021) 153:e282–9. 10.1016/j.wneu.2021.06.113 34217857

[17] Li J Yuan Y Liao X Yu Z Li H Zheng J. Prognostic significance of admission systemic inflammation response index in patients with spontaneous intracerebral hemorrhage: a propensity score matching analysis. Front Neurol. (2021) 12:718032. 10.3389/fneur.2021.718032 34630289 PMC8497988

[18] Zhang Y Xing Z Zhou K Jiang S. The predictive role of systemic inflammation response index (SIRI) in the prognosis of stroke patients. Clin Interv Aging. (2021) 16:1997–2007. 10.2147/CIA.S339221 34880606 PMC8645951

[19] Kelly PJ Lemmens R Tsivgoulis G. Inflammation and stroke risk: a new target for prevention. Stroke. (2021) 52:2697–706. 10.1161/STROKEAHA.121.034388 34162215

[20] Soehnlein O Libby P. Targeting inflammation in atherosclerosis - from experimental insights to the clinic. Nat Rev Drug Discov. (2021) 20:589–610. 10.1038/s41573-021-00198-1 33976384 PMC8112476

[21] Bäck M Yurdagul A Tabas I Öörni K Kovanen PT. Inflammation and its resolution in atherosclerosis: mediators and therapeutic opportunities. Nat Rev Cardiol. (2019) 16:389–406. 10.1038/s41569-019-0169-2 30846875 PMC6727648

[22] Spagnoli LG Mauriello A Sangiorgi G Fratoni S Bonanno E Schwartz RS. Extracranial thrombotically active carotid plaque as a risk factor for ischemic stroke. JAMA. (2004) 292:1845–52. 10.1001/jama.292.15.1845 15494582

[23] Denorme F Portier I Rustad JL Cody MJ de Araujo CV Hoki C. Neutrophil extracellular traps regulate ischemic stroke brain injury. J Clin Invest. (2022) 132:e154225. 10.1172/JCI154225 35358095 PMC9106355

[24] Garcia-Bonilla L Brea D Benakis C Lane DA Murphy M Moore J. Endogenous protection from ischemic brain injury by preconditioned monocytes. J Neurosci. (2018) 38:6722–36. 10.1523/JNEUROSCI.0324-18.2018 29946039 PMC6067076

[25] Chauhan A Al Mamun A Spiegel G Harris N Zhu L McCullough LD. Splenectomy protects aged mice from injury after experimental stroke. Neurobiol Aging. (2018) 61:102–11. 10.1016/j.neurobiolaging.2017.09.022 29059593 PMC5947993

[26] Chapman KZ Dale VQ Dénes A Bennett G Rothwell NJ Allan SM. A rapid and transient peripheral inflammatory response precedes brain inflammation after experimental stroke. J Cereb Blood Flow Metab. (2009) 29:1764–8. 10.1038/jcbfm.2009.113 19654587

[27] Xu S Lu J Shao A Zhang JH Zhang J. Glial cells: role of the immune response in ischemic stroke. Front Immunol. (2020) 11:294. 10.3389/fimmu.2020.00294 32174916 PMC7055422

[28] Tang Y Xu H Du X Lit L Walker W Lu A. Gene expression in blood changes rapidly in neutrophils and monocytes after ischemic stroke in humans: a microarray study. J Cereb Blood Flow Metab. (2006) 26:1089–102. 10.1038/sj.jcbfm.9600264 16395289

[29] Westendorp WF Dames C Nederkoorn PJ Meisel A. Immunodepression, infections, and functional outcome in ischemic stroke. Stroke. (2022) 53:1438–48. 10.1161/STROKEAHA.122.038867 35341322

[30] Muir KW Weir CJ Alwan W Squire IB Lees KR. C-reactive protein and outcome after ischemic stroke. Stroke. (1999) 30:981–5. 10.1161/01.STR.30.5.981 10229731

[31] Smedbakken L Jensen JK Hallén J Atar D Januzzi JL Halvorsen B. Activated leukocyte cell adhesion molecule and prognosis in acute ischemic stroke. Stroke. (2011) 42:2453–8. 10.1161/STROKEAHA.110.612440 21757661

[32] Cheng X Lian Y-J Ma Y-Q Xie N-C Wu C-J. Elevated serum levels of CXC chemokine ligand-12 are associated with unfavorable functional outcome and mortality at 6-month follow-up in chinese patients with acute ischemic stroke. Mol Neurobiol. (2017) 54:895–903. 10.1007/s12035-015-9645-9 26780459

[33] Wang C Gao L Zhang Z-G Li Y-Q Yang Y-L Chang T. Procalcitonin Is a stronger predictor of long-term functional outcome and mortality than high-sensitivity C-reactive protein in patients with ischemic stroke. Mol Neurobiol. (2016) 53:1509–17. 10.1007/s12035-015-9112-7 25650122

[34] Richard S Lagerstedt L Burkhard PR Debouverie M Turck N Sanchez J-C. E-selectin and vascular cell adhesion molecule-1 as biomarkers of 3-month outcome in cerebrovascular diseases. J Inflamm. (2015) 12:61. 10.1186/s12950-015-0106-z 26543408 PMC4634720

[35] Frøyshov HM Bjørnerem Å Engstad T Halvorsen DS. Elevated inflammatory markers predict mortality in long-term ischemic stroke-survivors: a population-based prospective study. Aging Clin Exp Res. (2017) 29:379–85. 10.1007/s40520-016-0575-9 27146666

[36] Bonifačić D Toplak A Benjak I TokmadŽić VS Lekić A Kučić N. Monocytes and monocyte chemoattractant protein 1 (MCP-1) as early predictors of disease outcome in patients with cerebral ischemic stroke. Wien Klin Wochenschr. (2016) 128:20–7. 10.1007/s00508-015-0878-4 26542133

[37] Soldozy S Yagmurlu K Norat P Elsarrag M Costello J Farzad F. Biomarkers predictive of long-term outcome after ischemic stroke: a meta-analysis. World Neurosurg. (2022) 163:e1–42. 10.1016/j.wneu.2021.10.157 34728391

[38] Chen C Gu L Chen L Hu W Feng X Qiu F. Neutrophil-to-lymphocyte ratio and platelet-to-lymphocyte ratio as potential predictors of prognosis in acute ischemic stroke. Front Neurol. (2020) 11:525621. 10.3389/fneur.2020.525621 33569032 PMC7868420

[39] Park M-G Kim M-K Chae S-H Kim H-K Han J Park K-P. Lymphocyte-to-monocyte ratio on day 7 is associated with outcomes in acute ischemic stroke. Neurol Sci. (2018) 39:243–9. 10.1007/s10072-017-3163-7 29086124

[40] Huang L. Increased systemic immune-inflammation index predicts disease severity and functional outcome in acute ischemic stroke patients. Neurologist. (2022). 10.1097/NRL.0000000000000464 36125980 PMC9812414

[41] Zhou Y Zhang Y Cui M Zhang Y Shang X. Prognostic value of the systemic inflammation response index in patients with acute ischemic stroke. Brain Behav. (2022) 12:e2619. 10.1002/brb3.2619 35588444 PMC9226852

